# CellMissy: a tool for management, storage and analysis of cell migration data produced in wound healing-like assays

**DOI:** 10.1093/bioinformatics/btt437

**Published:** 2013-08-05

**Authors:** Paola Masuzzo, Niels Hulstaert, Lynn Huyck, Christophe Ampe, Marleen Van Troys, Lennart Martens

**Affiliations:** ^1^Department of Medical Protein Research, VIB and ^2^Faculty of Medicine and Health Sciences, Department of Biochemistry, Ghent University, Ghent 9000, Belgium

## Abstract

**Summary:** Automated image processing has allowed cell migration research to evolve to a high-throughput research field. As a consequence, there is now an unmet need for data management in this domain. The absence of a generic management system for the quantitative data generated in cell migration assays results in each dataset being treated in isolation, making data comparison across experiments difficult. Moreover, by integrating quality control and analysis capabilities into such a data management system, the common practice of having to manually transfer data across different downstream analysis tools will be markedly sped up and made more robust. In addition, access to a data management solution creates gateways for data standardization, meta-analysis and structured public data dissemination.

We here present CellMissy, a cross-platform data management system for cell migration data with a focus on wound healing data. CellMissy simplifies and automates data management, storage and analysis from the initial experimental set-up to data exploration.

**Availability and implementation:** CellMissy is a cross-platform open-source software developed in Java. Source code and cross-platform binaries are freely available under the Apache2 open source license at http://cellmissy.googlecode.com.

**Contact:** lennart.martens@ugent.be

**Supplementary Information:** Supplementary data are available at *Bioinformatics* online.

## 1 INTRODUCTION

**Fig. 1.**
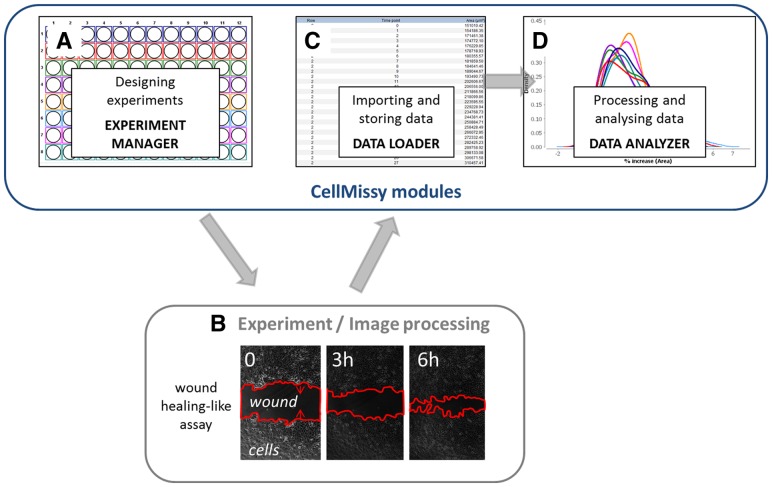
Schematic workflow of a typical wound healing experiment demonstrating the tasks for which the different modules (**A**, **C** and **D**) from CellMissy are used to manage, import and analyze data of images that are acquired independently by an experimental set-up and image processing tool of choice (**B**)

Dynamic cell motility processes are essential for morphogenesis, immune responses, wound healing and cancer metastasis (Friedl *et al.*, 2009). Investigation of the mechanisms and modes of cell migration is therefore important for fundamental scientific insight and for translational research, as molecules involved in deregulated cell migration are attractive targets for therapeutic intervention. As a result, the field has developed methods for the systematic high-throughput analysis of cell migration, with wound healing-like assays currently most amenable to multiple condition comparison or high-throughput approaches (Ashby *et al.*, 2012; Hulkower *et al.*, 2011). In these assays, a cell layer is allowed to move into a cell-free region, and the decrease in time of this area reflects cell migration velocity. The cell-free zone can be created (Riahi *et al.*, 2012) by scratching a confluent cell layer [typical wound healing assay (Liang *et al.*, 2007)] or by using non-damaging barriers during cell seeding [cell exclusion zone assay (Gough *et al.*, 2011)]. Advanced microscopes then allow automatic acquisition of images of the migrating cells, and specialized image processing tools are used to quantify cell migration features from these images (Table 1). Specific solutions for tackling the problems in large-scale image management and storage have additionally been reported (de Chaumont *et al.*, 2012; Moore *et al.*, 2008). However, a major current challenge in the cell migration field lies in the development of suitable software for the annotation, management and sharing of the quantification data obtained from such imaging-based experiments. Although software tools are available to help with several of the steps in this workflow, several key functions currently remain unmet (see Table 1). We here present CellMissy, a cross-platform system for the annotation, storage, analysis and dissemination of quantitative cell migration data. It aids the user with experimental set-up and annotation, generic data import/export (downstream from image processing) and subsequent data storage, inspection and analysis. It thus provides support for the entire experimental workflow, when coupled to an image analysis algorithm of choice (Fig. 1, Table 1). CellMissy is intended to facilitate local storage and analysis of acquired data, but it also provides the basic infrastructure for improved annotation, standardization and dissemination in the migration field, ultimately enabling re-analyses and meta-analyses to take place. The benefit of the latter has already been proven in other fields, where data management and dissemination infrastructure are more mature (Matic *et al.*, 2012; Martens, 2011). The development of user-oriented freely available data storage and analysis systems for cell migration data thus provides an important and timely next step for the field.

**Table 1.** Overview of currently available tools/software dedicated to wound healing-like workflow

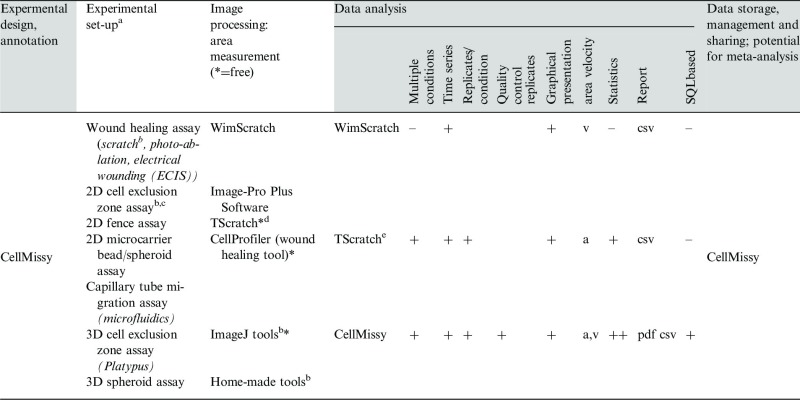

*Note:* The table headers show the typical steps in a wound healing-like workflow; within gray are those to which CellMissy contributes. The first column under ‘Data analysis’ gives the software; ‘–’, ‘+’ or ‘++’ indicates absence or level of presence of the listed feature; file format of report is indicated.

^a^Common conditional feature: migration of (confluent) cell layer followed in time (Kramer *et al.*, 2013).

^b^used as input in current work, but any of these set-ups can provide input to CellMissy.

^c^various set-ups (from Platypus, Ibidi, Cell Biolabs).

^d^Gebäck *et al.*, 2009.

## 2 TOOL DESCRIPTION AND FUNCTIONALITY

CellMissy is a client-server application with a graphical user interface (GUI) on the client and a relational database in the back-end to store the data (Supplementary Fig. S1). It is developed to follow the steps typically encountered in a cell migration experiment (Fig. 1 and Table 1) and is composed of three modules: the experiment manager, the data loader and the data analyzer. CellMissy handles wound healing-like data obtained from different experimental set-ups and after processing with different image analysis software (see Table 1). CellMissy takes either generic text files as input or can be customized to load data from image acquisition software automatically. CellMissy is written entirely in Java, and uses Swing for the GUI, the Spring framework for the logic layer and Hibernate for communication with the database.

### 2.1 Experiment manager–experimental set-up

This module (Fig. 1A) is used to set up a cell migration experiment. Its main interface element is a graphical presentation of a user-definable multi-well plate. For a certain biological condition, one or more wells representing technical replicates can be selected. Different informative metadata variables (e.g. cell line, treatment compound and dose) can be supplied for each condition. The resulting plate layout, well assignments and metadata are all stored in the database. To our knowledge, CellMissy constitutes the first tool in the community that provides the possibility to extensively annotate and customize the experimental set-up. CellMissy can also export the experimental design in PDF form for use in the laboratory, to actually set up the experiment and proceed to sample preparation. Furthermore, it is possible to retrieve set-up settings from previously stored experiments, thus accelerating the process of planning new experiments or setting up a replicate experiment. Templates containing all metadata information from the set-up can in addition be exported as an XML file, which can be shared between researchers and re-imported, thus allowing for comparison between different set-ups and promoting data exchange and dissemination.

### 2.2 Data loader–data import and storage

As shown in Figure 1B, the user then performs laboratory-dedicated image acquisition and processing (see Table 1 for choice set-ups and image processing tools), thus producing the input data for CellMissy (see Supplementary Section S2.1). Data import and storage are handled by the data loader module (Fig. 1C) that either reads a generic text-based data input format or can be fully automated if tailored to the chosen combination of set-up/image processing tool. CellMissy expects at least one tab-separated text file for each well, listing the time and the associated area values. Multiple files can be loaded *per* well, allowing different imaging locations, the application of different imaging techniques (e.g. phase contrast, fluorescence) or the import of data from different image analysis algorithms for that single well. Importantly, CellMissy again provides a means to export an entire experiment to an XML file at this level, i.e. with all acquired data. This XML file can be shared with other researchers (e.g. as Supplementary Information), directly re-imported by any CellMissy user, thus enabling easy exchange of data, re-analysis and reproduction of the results.

### 2.3 Data analyzer–data interpretation and reporting

The data analyzer module (Fig. 1D) guides the user through data analysis and interpretation. Table 1 demonstrates the extra features of CellMissy compared with the two reported tools dedicated to wound healing data analysis. The analysis in TScratch (Gebäck *et al.*, 2009) is an extension of the TScratch image processing, whereas CellMissy uniquely combines more extensive analysis of wound healing with data storage and management and possibilities for data dissemination (see above). The analysis consists first of a pre-processing step to normalize raw data relative to the area at time 0 (see Supplementary Section S2.1). The next step handles quality control (for full details, see Supplementary Section S2.2). For each technical replicate, artifacts in area increase between time steps are detected and visualized using a kernel density estimator (Supplementary Fig. S5). The variation between technical replicates is examined using Euclidean distance (Supplementary Fig. S6). In each step, the user can accept or reject the suggested correction. A linear regression model then extracts slope and *R*^2^ value on a replicate level. The median slope across replicates then yields the median migration velocity for a condition. All pair-wise differences in median velocity between a set of user-selected conditions are subsequently analyzed using a Mann–Whitney *U* test with a choice of multiple hypothesis testing correction (Bonferroni or Benjamini–Hochberg) (Supplementary Section S2.3). The architecture of CellMissy is, in addition, fully pluggable for analysis algorithms. This means that different distance metrics, statistical tests, kernel density estimators, outlier detection methods and multiple hypothesis correction methods can be added by any interested developer. Such new methods can be plugged dynamically into CellMissy, and will subsequently be immediately available to the user from the CellMissy interface. Finally, CellMissy produces a detailed PDF report that summarizes experimental design details, acquired data, corresponding results and statistical evaluation.

## 3 CONCLUSIONS

CellMissy is a novel, cross-platform, generic data management and analysis system for cell migration experiments. CellMissy is freely available, flexible in linking to upstream experimental set-ups and image processing tools, easily extensible and automates data handling, processing, quality monitoring and interpretation. Its intuitive interface provides support from the experimental set-up to the final reporting of the analysis including statistics. Data in CellMissy can be exported from one system and re-loaded in another system, enabling easy data sharing. In addition, it provides a framework for tool expansion toward future meta-analyses on data generated by multiple users, making it possible, for example, to compare all drug effects on cells of a particular cancer present in a shared CellMissy database. CellMissy is released as open source under the permissive Apache2 license.

## Supplementary Material

Supplementary Data
