# Supplementary material for: CellMissy: a tool for management, storage and analysis of cell migration data produced in wound healing-like assays
Source: Bioinformatics. 2013 Aug 5;29(20):2661–3. doi: 10.1093/bioinformatics/btt437 (PMC3789541; doi:10.1093/bioinformatics/btt437)
Supplement: Supplementary Data [file supp_29_20_2661__index.html]

CellMissy: a tool for management, storage and analysis of cell migration data produced in woundhealing-like assays. — CellMissy: a tool for management, storage and analysis of cell migration data produced in wound healing-like assays — CellMissy: a tool for management, storage and analysis of cell migration data produced in wound healing-like assays — Supplementary Data 

# CellMissy: a tool for management, storage and analysis of cell migration data produced in wound healing-like assays

## Supplementary Data

files

**Files in this Data Supplement:**

- Supplementary Data - docx file
