# Supplementary material for: CellMissy: a tool for management, storage and analysis of cell migration data produced in wound healing-like assays
Source: Bioinformatics. 2013 Aug 5;29(20):2661–3. doi: 10.1093/bioinformatics/btt437 (PMC3789541; doi:10.1093/bioinformatics/btt437)
Supplement: Supplementary Data [file supp_btt437_Masuzzo_CellMissy_Supplementary.docx]

# Supplementary Data for

# CellMissy: a tool for management, storage and analysis of cell migration data produced in wound healing-like assays.

Paola Masuzzo^1, 2^, Niels Hulstaert^1, 2^, Lynn Huyck^1^, Christophe Ampe^1^, Marleen Van Troys^1,#^ and Lennart Martens^1, 2,#,*^^[[1]](#footnote-1)^

^1^ Department of Biochemistry, Faculty of Medicine and Health Sciences, Ghent University, Ghent, Belgium

^2^ VIB Department of Medical Protein Research, Ghent University, Ghent, Belgium

## S1 - CellMissy Database Schema


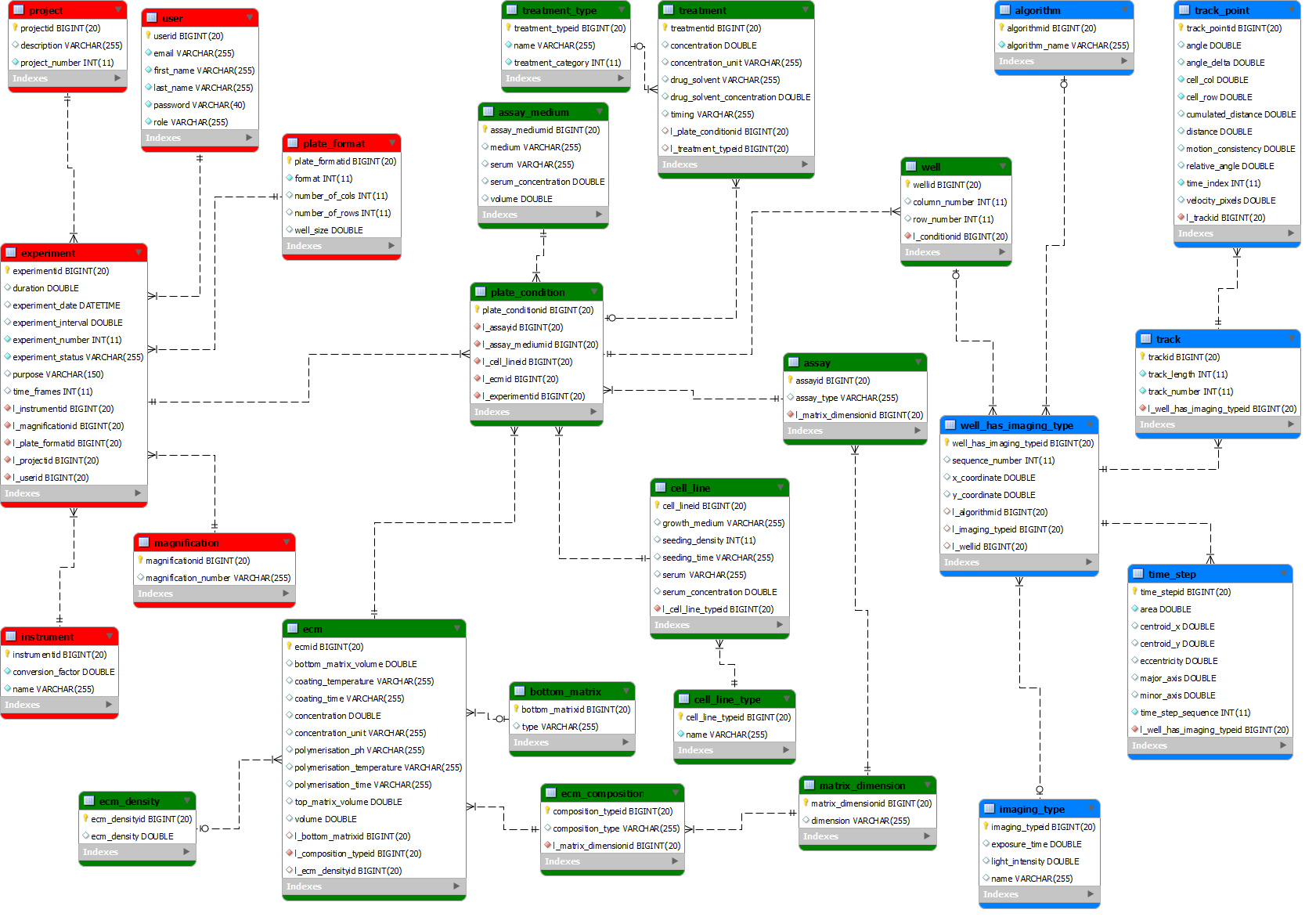


Fig. S1 Schema of the CellMissy relational database: project/experiment metadata (red tables), biological condition metadata (green tables), and cell migration data (blue tables).

## S2 - Tool functionality: investigating a wound healing cell migration experiment

### Versatility in Data Loading

### Cell-covered area *vs* open area

When investigating cell migration with common wound healing-like assays (scratch and cell exclusion zone assays or others, see Table 1 in main text), data readouts can be based on the cell-covered area (area increase over time) or on the open area, i.e. the area of the initially cell-free gap that eventually becomes covered with cells (area decrease over time) (see Fig. S2 below for a representation of these different measured areas). In the Data Analyzer module of CellMissy, the user can choose to import and work with both types of measured area, in order to proceed with data analysis and investigation. In all cases, data are always normalized in terms of the area measurement at the time frame of the start. For the open area values, the normalization is done so that the first values of each technical replicate are equal to 100% (full gap area) and values are then expressed in percentage. These open area values are subsequently converted to cell-covered area values (values complementary to 100%). Fig. S2 shows this procedure for data obtained for images from a scratch assay (biological condition: mouse embryonic fibroblast cell line, untreated, three technical replicates; 12 h imaging, interval 30 min), for which the Manual Tracking plugin image processing tools of ImageJ was used to delineate the open (wound) area values. Txt files with open area in time for the three replicates were loaded in CellMissy (see 1.2).


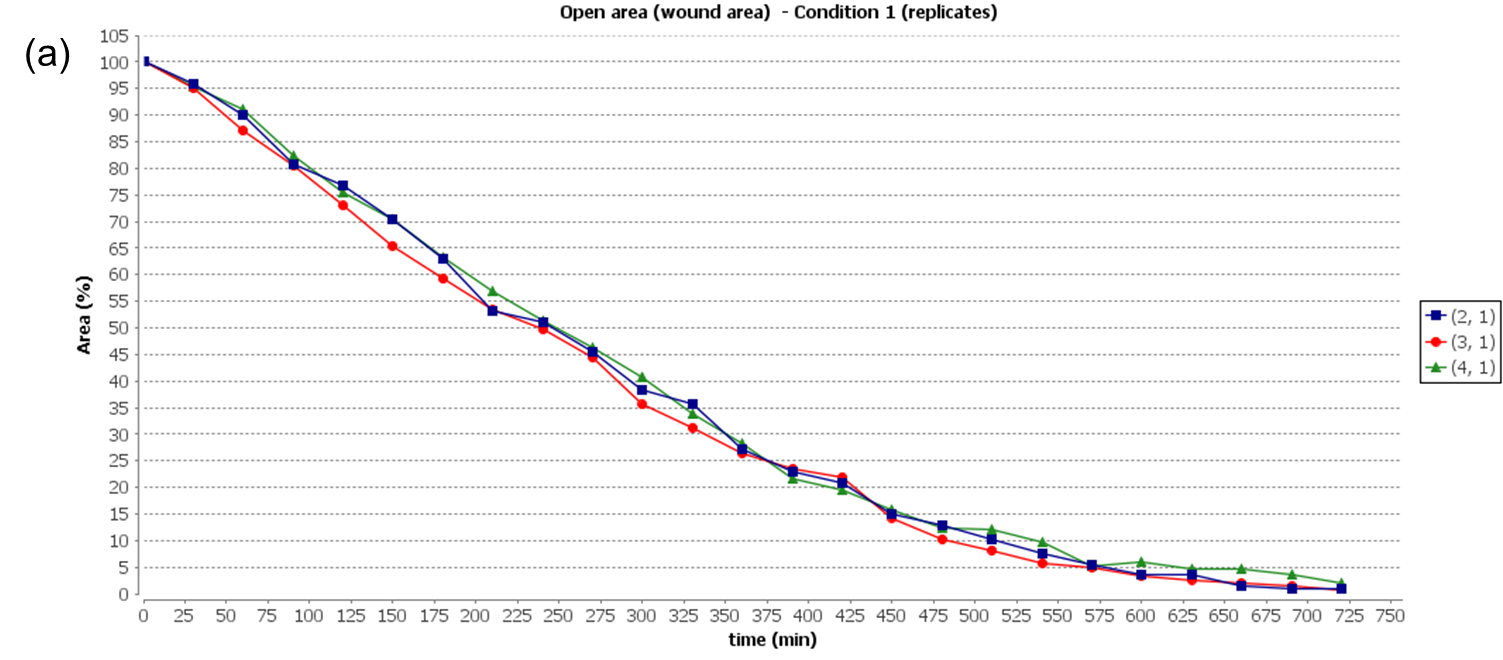


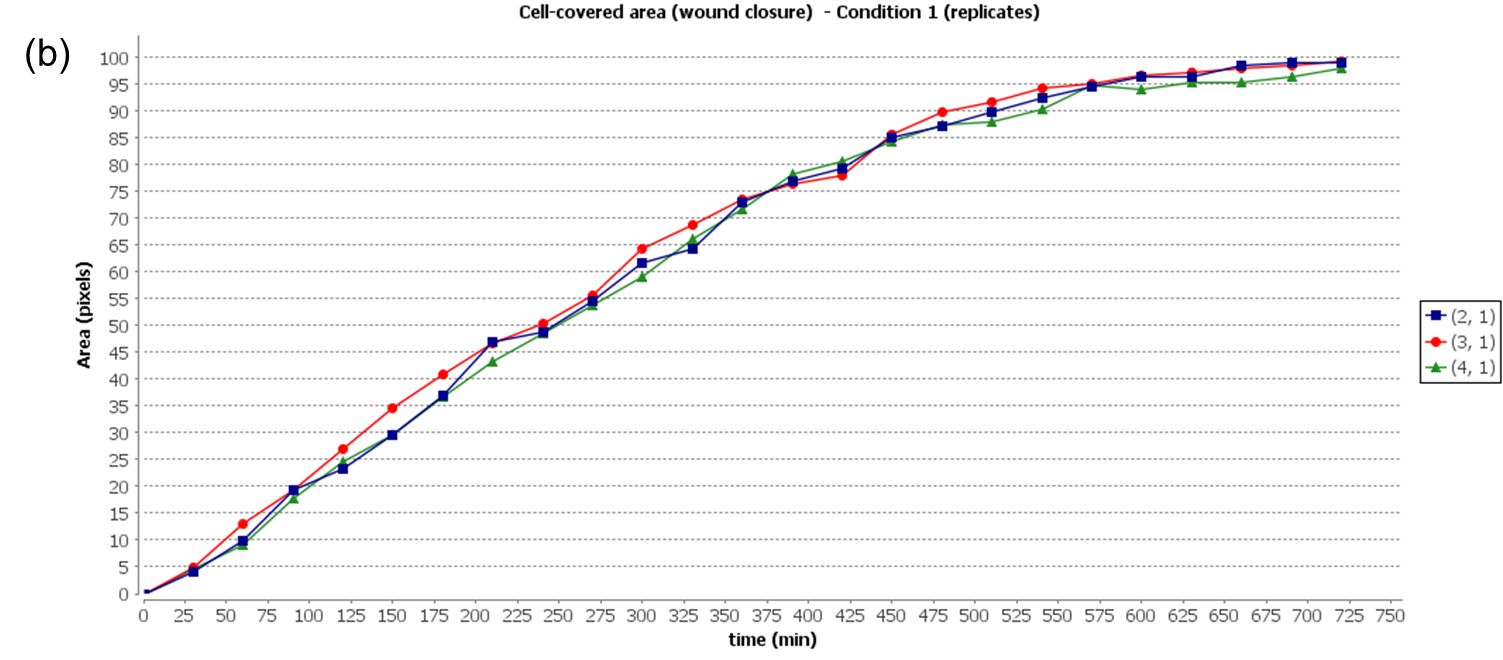


**Fig. S2** Example of evolution in time of area values from a scratch assay. **(a)** Open area values for three technical replicates of a biological condition normalized to starting value of 100%. **(b)** The same data are plotted after conversion to cell-covered area values. Calculation of migration velocity of cells in CellMissy is based on the cell-covered area increase in time as shown in the lower graph.

### 1.2 Generic input format and automated data loading

Data import and storage in CellMissy uses the generic migration tracking input format described in the main text and here shown in Fig. S3. Alternatively, CellMissy also provides automated data loading for the migration set-up used in our group, i.e. an Olympus xcellence system (CellM) and dedicated imaging processing software (Huyck et al., *in preparation*). As explained in more detail on <http://cellmissy.googlecode.com>, the automated loading exploits metadata generated by the microscope (*in casu* obsep file) and the image acquisition software, as well as stored information of imaging order of the wells. As such, the automated loading capacity of CellMissy could – by addition of an extra adapter code - be customized to work in principle with any microscopy image acquisition-processing combination.


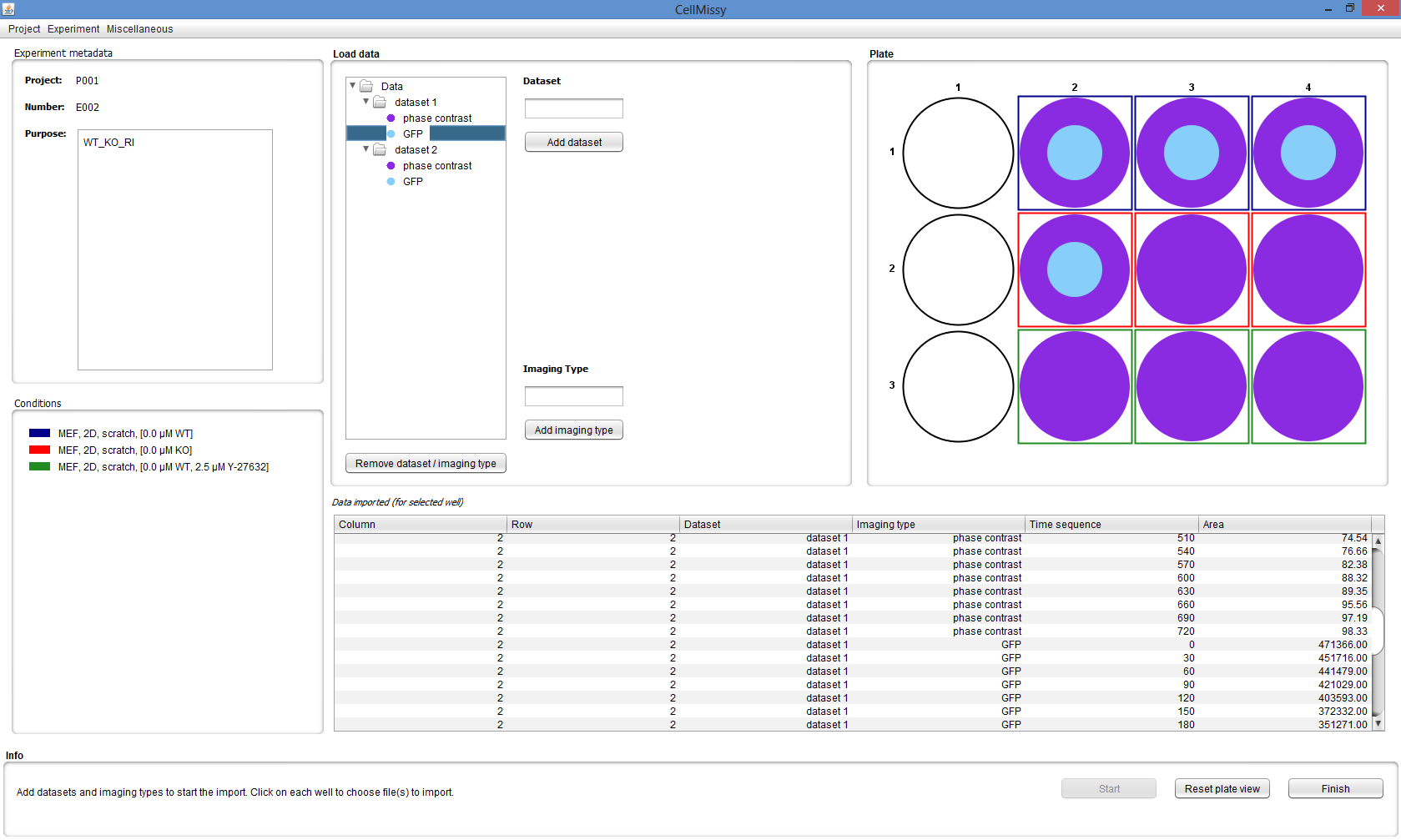


**Fig. S3** Screenshot of the Data Loader in CellMissy using the generic migration data input format. As in each module of CellMissy, the plate view with the biological annotated conditions is reported. The user provides experimental metadata (number of time frames and interval between measurements), and assigns text files with data to a certain well. Per sample multiple data sets can be loaded: data from different imaging techniques (e.g. phase contrast (purple circles) and fluorescence (light blue circles)) or from different image analysis set-ups (e.g. different algorithms settings during image processing).

### Data quality control

When fully automated image processing packages are used, it is our experience that artifacts may occur in the delineation of the cell-covered or open area, especially in phase contrast imaging. These errors in segmentation may be due to experimental issues (cells or non-cell particles in the open area), too low contrast to detect and correctly segment the cell-covered area in some images (leading thus to temporary underestimation of the area) etc. Especially for high-throughput experiments, correction for segmentation errors is therefore essential during down-stream data analysis. In this context, CellMissy provides tools for unbiased data quality control, as described in the next sections. The example shown in Fig. S2 clearly demonstrates that for data based on manual segmentation of the area, correction is less required and CellMissy consequently also provides the possibility to retain the raw data and ignore these corrections.

#### 2.1 Outlier visualization in area increase with Kernel Density Estimation

To correct for possible artificial changes in area due to image segmentation errors, CellMissy calculates *per* well (i.e. *per* replicate of a biological condition) the area increase and % area increase between the consecutive time steps. Subsequently, the distribution of all % area increases for a well is considered. More in detail, a Kernel Density Estimator (KDE) is implemented to evaluate the probability density function of the % area increase during wound closure between consecutive time points. A normal kernel function is used (even though a different function can be easily plugged in CellMissy) and the optimal bandwidth is computed through Silverman's ‘rule of thumb’. A % area increase is detected as an outlier if it falls outside the range:

$$[Q_{1}-1.5 \left( Q_{3}-Q_{1} \right),Q_{3}+1.5 \left( Q_{3}-Q_{1} \right)]$$

with *Q_1_* and *Q_3_* the lower and the upper quartile of the distribution, respectively. Fig. S4 shows an example of outlier visualization in CellMissy using a KDE.


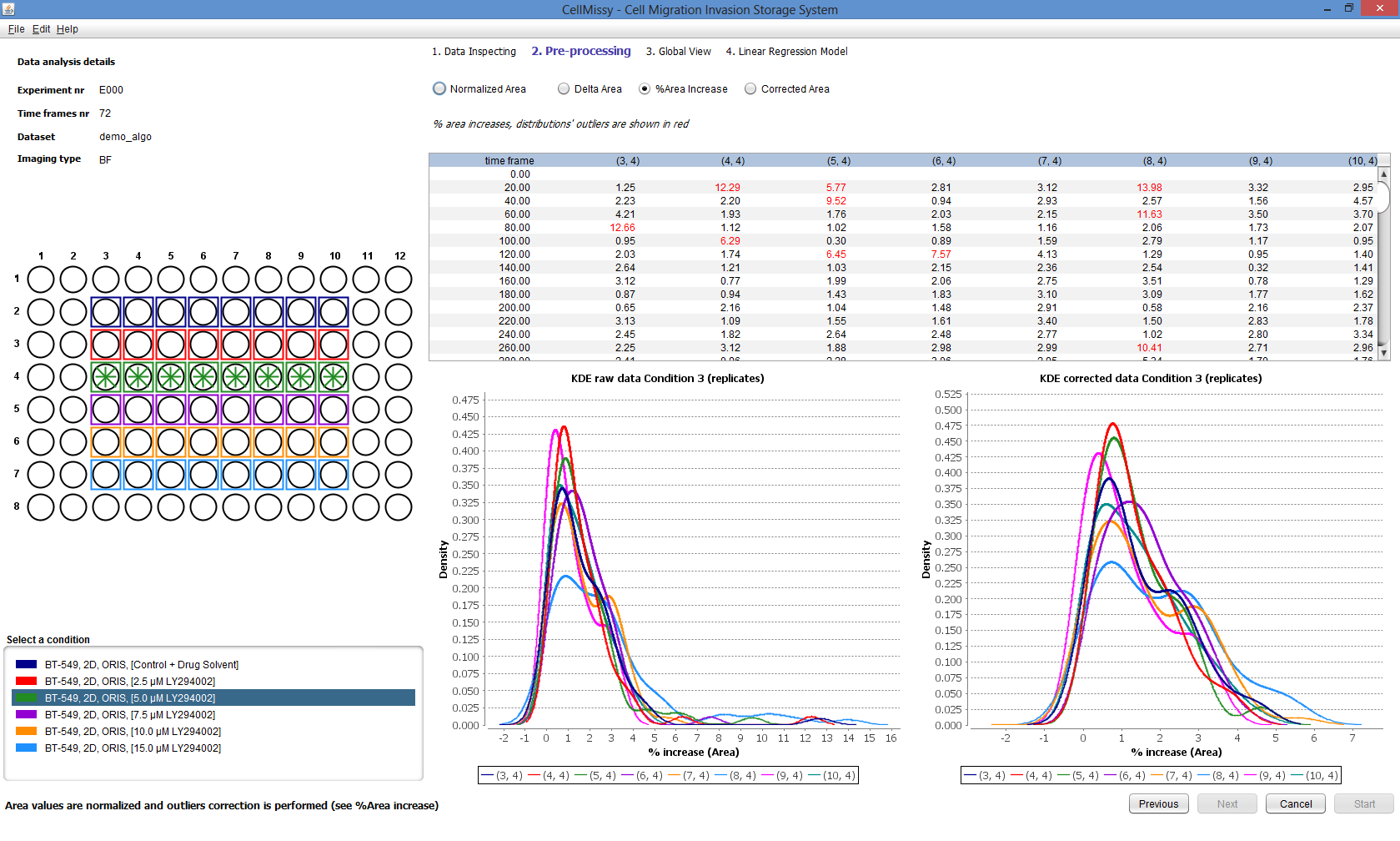


**Fig. S4** The probability density functions of the area increase between consecutive time points are plotted for each replicate of biological condition 3 (wells are marked with stars in the plate layout). Outliers present in the raw data (left chart, outliers highlighted in red in table) are removed after correction (right chart).

#### 2.2 Technical precision assessment with Euclidean distance

The previous section detailed on quality control on the level of one technical replicate (well). In a typical wound healing experiment several replicates are run for a specific condition. Putative technical mistakes or image processing errors may make it opportune that not all replicates within one biological condition are considered in the analysis. CellMissy allows the identification of outliers in a set of technical replicates. Let *n* be the number of technical replicates in a biological condition and *m* the number of time points for which the evolution of the area has been followed, then the dataset *per* biological condition is characterized as a collection of *n* sample vectors in an *m*-dimensional space. The Euclidean distance between two sample vectors (i.e. two replicates) *x* and *y* is taken as a metric to assess their similarity:

$$d\left( x, y \right)= \sqrt{\sum_{i=1}^{m} {(x_{i}-y_{i})}^{2}}$$

Please note that a different distance metric can be used as well, as soon as it’s plugged into CellMissy. The Euclidean distance matrix containing the *n*^2^ pairwise distances between the replicates is first computed, and then outlier detection is carried out using the abovementioned interquartile range criterion *per* matrix row (i.e. *per* replicate). A replicate (sample vector) is detected as an outlier, and can therefore be excluded from the replicate set, if more than 50% of its distances to other replicates are outliers. This automatic classification among technical replicates ensures an objective, deterministic data quality control. However, the user is allowed to override the outlier removal suggestion made by CellMissy. Fig. S5 shows an example of data quality control in CellMissy on technical replicates level.


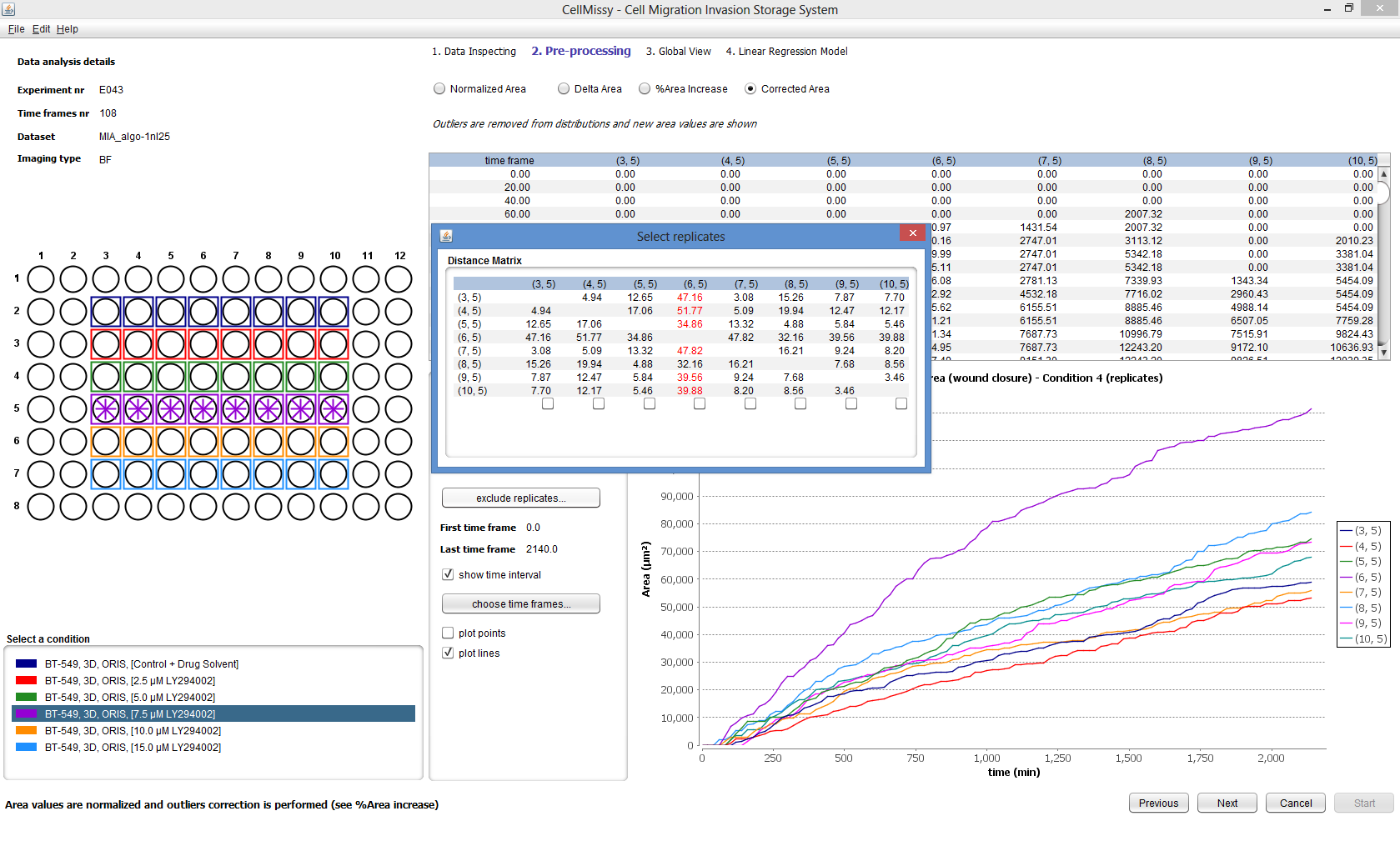


**Fig. S5** Technical precision is analyzed for biological condition 4 (wells are marked with stars in the plate layout) using Euclidean distance. Technical replicate in well (6, 5) (purple in the chart) is detected as outlier (highlighted in red in table).

### Data interpretation and statistical analysis

CellMissy computes the median area values in time across all replicates for each biological condition, as shown in Fig. S6, providing a first, visual estimation of the area change in time for the experimental conditions.


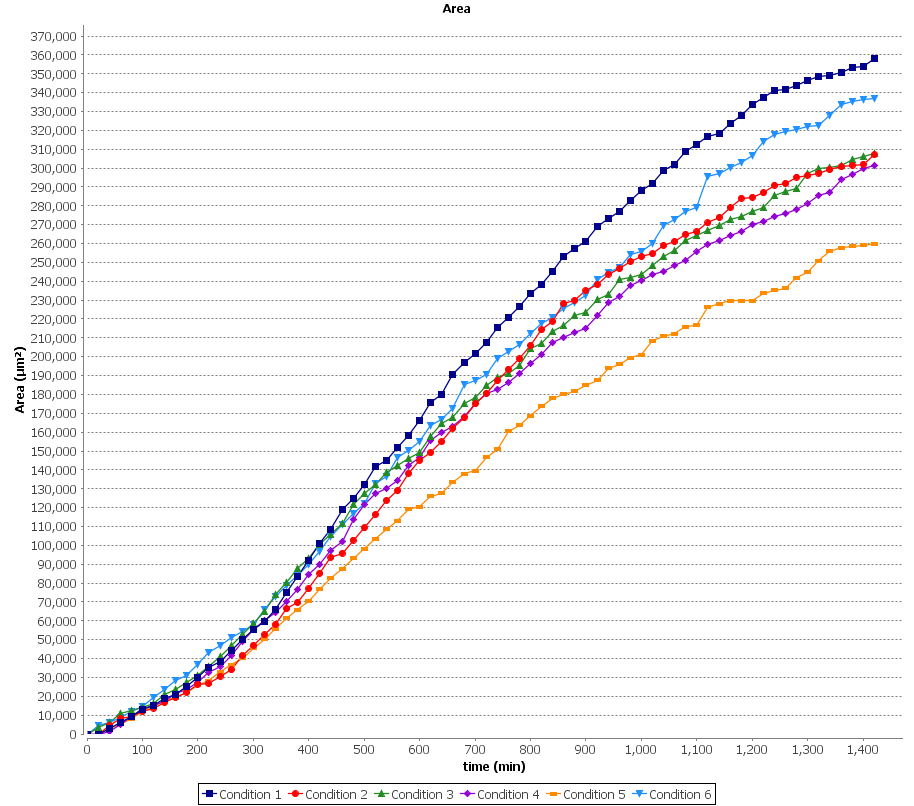


Fig. S6 Evolution of the median cell-covered area over time for six biological conditions of an experiment.

CellMissy then makes use of a linear regression model to extract a slope and R^2^ of the area over time for each replicate in a biological condition. The median slope across the replicates is then calculated to provide the median velocity with which the cells close the wound or the cell free zone. Given a set of user-selected biological conditions, a summary statistic is provided for each of these conditions, and all pair-wise differences in median velocity are analyzed using a Mann-Whitney U test; either Bonferroni or Benjamini-Hochberg can be chosen for multiple hypotheses testing correction. Different statistical tests and different multiple testing correction methods can be plugged into the tool. Fig. S7 shows parts of CellMissy interface in this step.


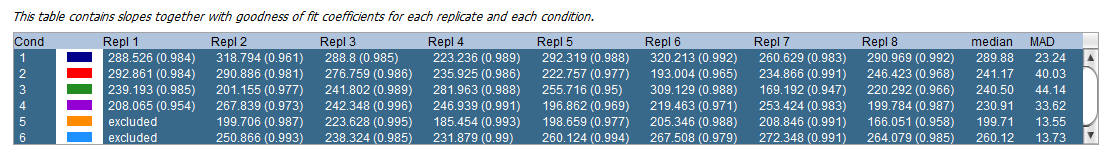


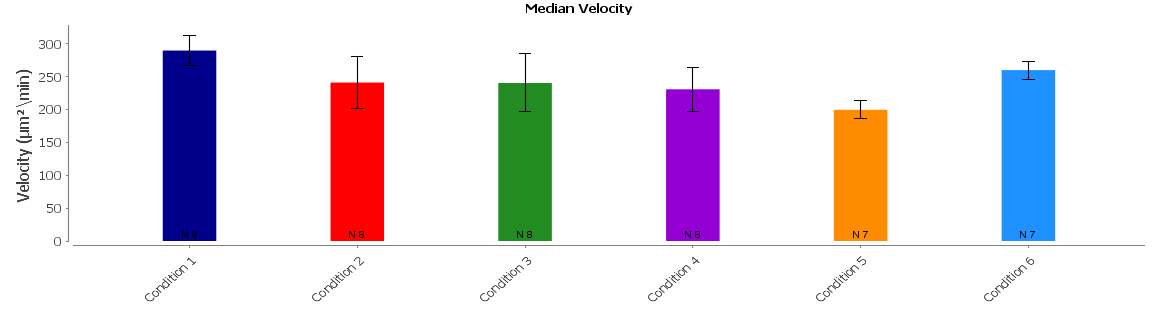


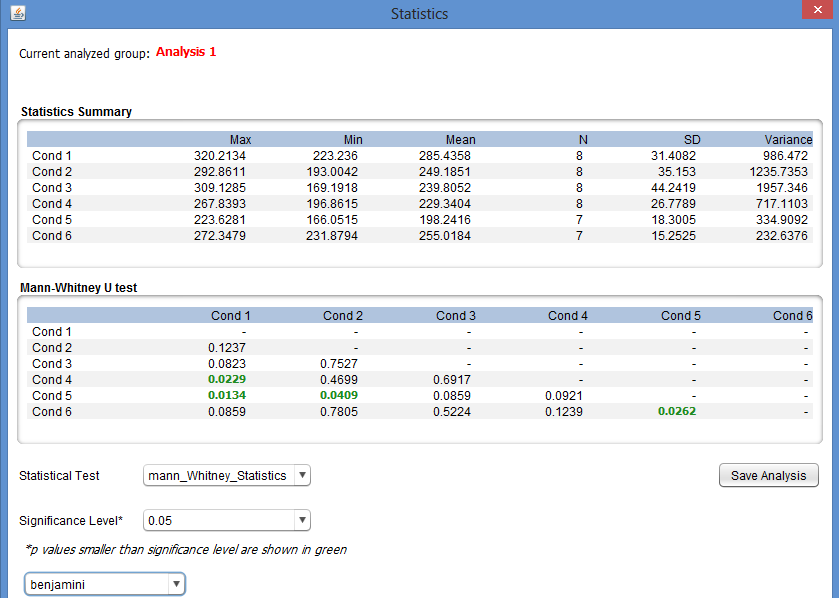


Fig. S7 Parts of the Data Analyzer module in CellMissy: table containing slopes and R² coefficients for each replicate, together with the bar chart with median velocity for each biological condition (N is the number of technical replicates) are shown at the top; summary statistics and p-values from a Mann-Whitney U test are presented in two distinct tables below. Note that significant differences are highlighted in green. Significance level and multiple correction method can be chosen by the user. Moreover, different statistical tests can be easily plugged-in, as well as algorithms for multiple testing correction.

1. ^#^ Equal contribution

   * To whom correspondence should be addressed [↑](#footnote-ref-1)
